# Effect of heparin treatment on pulmonary embolism and in-hospital death in unvaccinated COVID-19 patients without overt deep vein thrombosis

**DOI:** 10.1186/s12959-022-00393-z

**Published:** 2022-06-20

**Authors:** Bruno Bais, Emanuela Sozio, Daniele De Silvestri, Stefano Volpetti, Maria Elena Zannier, Carla Filì, Flavio Bassi, Lucia Alcaro, Marco Cotrufo, Alberto Pagotto, Alessandro Giacinta, Vincenzo Patruno, Andrea Da Porto, Rodolfo Sbrojavacca, Francesco Curcio, Carlo Tascini, Leonardo Alberto Sechi, GianLuca Colussi

**Affiliations:** 1 Thrombosis Prevention Unit, Division of Internal Medicine, Academic Hospital of Udine (ASUFC), 33100 Udine, UD Italy; 2 Infectious Diseases Clinic, Department of Medicine, University of Udine, 33100 Udine, Italy; 3 Hematology and Bone Marrow Transplantation, Department of Medicine, University of Udine, 33100 Udine, Italy; 4 Department of Anesthesia and Intensive Care Medicine, Academic Hospital of Udine (ASUFC), 33100 Udine, Italy; 5 Internal Medicine, Department of Medicine, University of Udine, 33100 Udine, Italy; 6 Division of Pulmonary Medicine, Academic Hospital of Udine (ASUFC), 33100 Udine, Italy; 7 Department of Laboratory Medicine, University of Udine, 33100 Udine, Italy

**Keywords:** Anticoagulant, Multi-state model, Survival analysis, Retrospective study, D-dimer

## Abstract

**Background:**

Pulmonary embolism (PE) without overt deep vein thrombosis (DVT) was common in hospitalized coronavirus-induced disease (COVID)-19 patients and represented a diagnostic, prognostic, and therapeutic challenge. The aim of this study was to analyze the prognostic role of PE on mortality and the preventive effect of heparin on PE and mortality in unvaccinated COVID-19 patients without overt DVT.

**Methods:**

Data from 401 unvaccinated patients (age 68 ± 13 years, 33% females) consecutively admitted to the intensive care unit or the medical ward were included in a retrospective longitudinal study. PE was documented by computed tomography scan and DVT by compressive venous ultrasound. The effect of PE diagnosis and any heparin use on in-hospital death (primary outcome) was analyzed by a classical survival model. The preventive effect of heparin on either PE diagnosis or in-hospital death (secondary outcome) was analyzed by a multi-state model after having reclassified patients who started heparin after PE diagnosis as not treated.

**Results:**

Median follow-up time was 8 days (range 1–40 days). PE cumulative incidence and in-hospital mortality were 27% and 20%, respectively. PE was predicted by increased D-dimer levels and COVID-19 severity. Independent predictors of in-hospital death were age (hazards ratio (HR) 1.05, 95% confidence interval (CI) 1.03–1.08, *p* < 0.001), body mass index (HR 0.93, 95% CI 0.89–0.98, *p* = 0.004), COVID-19 severity (severe versus mild/moderate HR 3.67, 95% CI 1.30–10.4, *p* = 0.014, critical versus mild/moderate HR 12.1, 95% CI 4.57–32.2, *p* < 0.001), active neoplasia (HR 2.58, 95% CI 1.48–4.50, *p* < 0.001), chronic obstructive pulmonary disease (HR 2.47; 95% CI 1.15–5.27, *p* = 0.020), respiratory rate (HR 1.06, 95% CI 1.02–1.11, *p* = 0.008), heart rate (HR 1.03, 95% CI 1.01–1.04, *p* < 0.001), and any heparin treatment (HR 0.35, 95% CI 0.18–0.67, *p* = 0.001). In the multi-state model, preventive heparin at prophylactic or intermediate/therapeutic dose, compared with no treatment, reduced PE risk and in-hospital death, but it did not influence mortality of patients with a PE diagnosis.

**Conclusions:**

PE was common during the first waves pandemic in unvaccinated patients, but it was not a negative prognostic factor for in-hospital death. Heparin treatment at any dose prevented mortality independently of PE diagnosis, D-dimer levels, and disease severity.

## Background

The coronavirus 2019 induced disease (COVID-19) is an interstitial pneumonia caused by severe acute respiratory syndrome coronavirus 2 (SARS-CoV-2) infection. The infection was endemic in Wuhan, China, in December 2019 and soon became responsible for the actual worldwide pandemic that is causing millions of deaths. In 2020–2021, Italy has been involved in three major waves of SARS-CoV-2 pandemics that comprised about 4.7 million infections and over 130,000 deaths [1]. Patients with severe COVID-19 were at high risk of pulmonary thrombosis, respiratory failure with resistance to oxygen treatment, and increased probability of organ support and in-hospital death [2]. Harrison et al. observed that venous thromboembolism (VTE) can be documented in 25% of patients hospitalized with COVID-19 and, in particular, up to 19% with pulmonary embolism (PE) and 7% with deep vein thrombosis (DVT) [3]. However, it is not clear whether all imaging described as pulmonary embolism resulted from a VTE process, since DVT was not commonly found in these patients [4].

Because of the elevated prothrombotic risk associated with COVID-19, societal indications suggested the use of heparin in the treatment of hospitalized patients [5]. According to the recent CHEST guideline and expert panel report for VTE prevention in COVID-19, critically ill patients should be treated with prophylactic doses of anticoagulants, whereas moderately ill patients, when not contraindicated, should start therapeutic doses [6]. This antithrombotic strategy was based upon recent multi-platform trails. In these trials the initial treatment with heparin at therapeutic dose regarding the standard thromboprophylaxis increased the survival probability of moderately ill patients admitted to the medical ward [7, 8], but in critically ill patients admitted to the intensive care unit (ICU) this approach was not effective and possibly harmful compared to the standard prophylaxis [8, 9]. Interestingly, the very recent meta-analysis of Flumignan et al. that included clinical trials and observational studies, questioned this approach by showing little to no difference in all-cause mortality of the high/therapeutic dose compared to the low/prophylactic dose of anticoagulants in all hospitalized COVID-19 patients, and confirmed an increased risk of bleeding with the higher doses [10]. Therefore, despite the improvement in our knowledge about the antithrombotic strategy for preventing VTE and mortality in COVID-19 patients, some questions remain unanswered. For example, it is uncertain the effective preventive dose of heparin for hospitalized patients according to disease severity [10], the role of a PE diagnosis on mortality [11–13], or the usefulness of d-dimer levels to guide timing and dosing of heparin treatment [14].

In this study, we presented the experience of a tertiary COVID-19 academic hospital during the first three waves of SARS-CoV-2 pandemic in the Northeast Italy. Because PE occurrence in patients without a documented DVT represents a diagnostic, prognostic, and therapeutic challenge, the aim of this study was to analyze the prognostic effect of PE on in-hospital mortality and the preventive role of different strategies of heparin treatment on PE and in-hospital mortality of unvaccinated COVID-19 patients without overt DVT.

## Patients and methods

### Study design and collected variables

This was a retrospective cohort study designed according to STROBE guidelines [15]. We collected data of COVID-19 patients admitted to the intensive care unit (ICU) or medical wards of the tertiary Academic Hospital of Udine from May 2020 to May 2021. We included consecutive patients unvaccinated for SARS-CoV-2 of all sexes, older than 18 years, with positive oropharyngeal swab samples for SARS-CoV-2 RNA, who have performed at least one computed tomography (CT) scan of the thorax with contrast enhancement, and were symptomatic for COVID-19. Patients who had an inconclusive SARS-CoV-2 molecular test result, were already anticoagulated at hospital admission, had ultrasound signs of DVT, were on dialysis, had contraindications to iodinated endovenous contrast enhancement, or had declined permission to use their data for research and pregnant women were excluded from the study. Severity of COVID-19 was classified at patient admission according to the World Health Organization (WHO) classification system in mild, moderate, severe, or critical disease [16]. All patients were treated with the best of medical knowledge according to WHO guidelines available during the study period [16].

Information about general clinical characteristics, anthropometric variables, vital signs, biochemical variables, and type of medications was extracted from the Hospital electronic database. In particular, the following variables were collected: age, sex, ICU admission, need for orotracheal intubation, body weight, height, comorbidities, body temperature, respiratory rate, arterial oxygen saturation, systolic blood pressure, heart rate, hemoglobin, white blood cell, lymphocyte, platelets count, plasma creatinine, C-reactive protein, and D-dimer levels. The body mass index (BMI) was calculated as weight in kilograms over the square of height in meters. The glomerular filtration rate was estimated by the Modification of Diet in Renal Disease (MDRD) study equation [17]. For relevant medications, we considered the use of angiotensin-converting enzyme inhibitors (ACEi), angiotensin II receptor blockers (ARB), statins, antiplatelet drugs, and heparin. Heparin dose was differentiated as “prophylactic” if the first prescription was equivalent or lower than 4000 international units (IU) of enoxaparin, or “intermediate/therapeutic” if greater than 4000 IU. Dose prescription included adjustment for body weight (BMI higher than 30 kg/m^2^), severe renal failure (eGFR lower than 30 ml/min/1.73 m^2^), and severe thrombocytopenia (platelets count lower than 50.000/mm^3^).

The primary outcome of the study was in-hospital death. The secondary outcome was PE diagnosis or in-hospital death in patients who started heparin to prevent PE events. Variables that predicted PE diagnosis and the effect of PE on the primary outcome were analyzed by a classical Cox survival model. In this model, any heparin use comprised the use of heparin for any reason (thromboprophylaxis or therapeutic) and time of initiation (before or after PE diagnosis). The preventive effect of heparin on the secondary outcome was analyzed using a time-dependent multi-state transition model. In this model, patients who started heparin after PE diagnosis were reclassified as not treated.

The Department of Medicine Institutional Review Board (IRB) of the University of Udine approved this study on November 16, 2021 (Protocol Number 087/2021). All patients signed a generic informed consent to use their data for research at hospital admission unless critically ill or deceased. The IRB stated that no additional specific informed consent was needed for the retrospective analysis of patients’ data.

### Laboratory methods

Molecular testing for SARS-CoV-2 infection on nasopharyngeal swab samples was performed by real-time reverse transcriptase polymerase chain reaction (RT-PCR) analysis of the virus RNA according to WHO guidelines [18]. CRP plasma levels were measured using a C-Reactive Protein gen.3 (CRPL3) assay on Cobas c 702® instrument (Roche). The test had a functional sensitivity of 1 mg/l. Plasma levels of the cross-linked fibrin degradation product D-dimer were measured by a latex enhanced immune-turbidimetric assay on an automated coagulation analyzer (ACL TOP, Instrumentation Laboratory). Results were reported as fibrinogen-equivalent units (FEU). All biochemical analyzes and other routine blood tests were performed in the certified laboratory service of the Academic Hospital of Udine. Analysis of blood gases and plasma bicarbonates concentration were performed with a point-of-care automated blood gas analyzer directly in the ICU or medical ward after patient admission.

### Lung imaging and venous ultrasound

All SARS-CoV-2-infected symptomatic patients underwent first-level lung imaging by ultrasound or plain radiography within 24 h of emergency department admission. Images suggestive of interstitial pneumonia or patients suspected for lung involvement independent of first-level lung imaging performed a computerized tomography (CT) scan with contrast enhancement and PE could be diagnosed, incidentally. A lung CT scan with contrast enhancement was repeated in those patients with worsen respiratory symptoms or arterial oxygen desaturation during follow-up. A compressive venous ultrasound examination of both inferior limbs, independent of clinical signs of DVT, was performed bedside in all patients with documented PE at CT-scan. Briefly, patients were positioned to maximize venous distension and B-mode images of the vein were observed with a transversal and longitudinal probe orientation. We performed a 2-point technique testing the compressibility of the common femoral vein and the popliteal vein. We used a probe of 7.0 MHz on a MyLab^TM^25Gold system (Esaote, Florence, Italy).

### Statistics methods

**Fig. 1:**
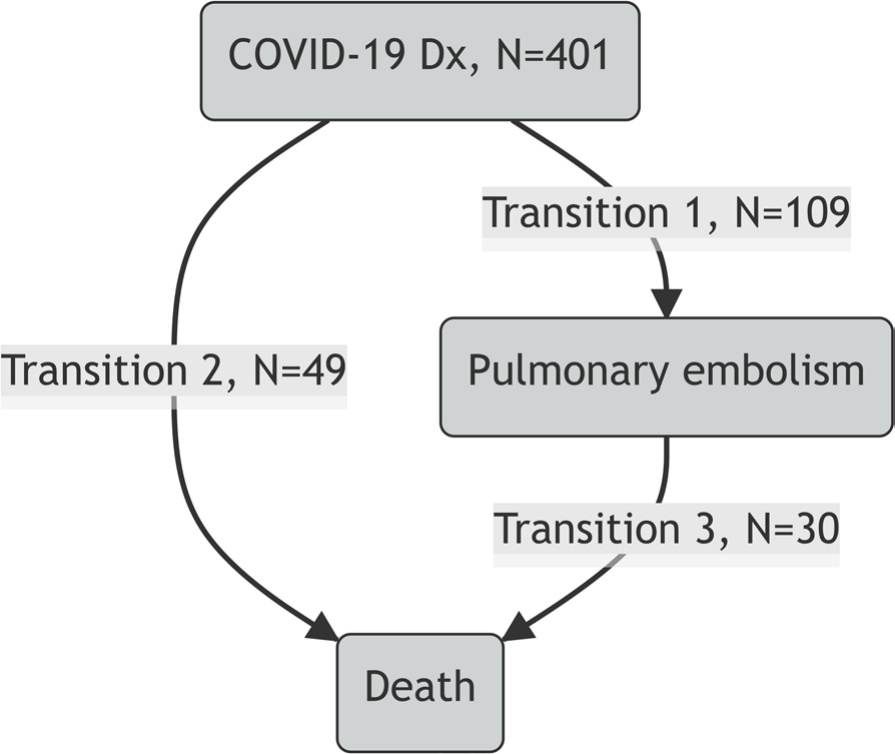
Multi-state transition model

Continuous variables were summarized as mean and standard deviation (SD) if normally distributed, or as median and interquartile range (IQR) if skewed. Normal distribution was assessed by looking at the histogram and performing the Shapiro–Wilk test. Categorical variables were summarized as count and percentage. The Student t-test was used for mean comparison of normal variables, whereas the non-parametric Mann–Whitney U test was used for skewed variables. In contingency tables, the Fisher exact test was used to compare frequencies. Patients in the mild or moderate WHO severity class were analyzed as a unique group. The survival probability was presented by the Kaplan–Meier curves. The unadjusted effect of PE diagnosis on survival probability was assessed by the non-parametric log-rank test. Variables that predicted PE diagnosis or the primary outcome were assessed by the univariate Cox analysis of proportional hazards. Multivariate analysis was used to assess which variable remained an independent predictor of PE diagnosis or of the primary outcome. In the multivariate models, we also included PE diagnosis and any heparin use variables, whichever probability they showed in univariate analysis. Multicollinearity was assessed by calculating the variance inflation factor for each variable in the multivariate model. Multicollinearity was significant when score was greater than 5 and variables that showed multicollinearity were dropped from the model [19]. To analyze the time dependent preventive effect of heparin on PE diagnosis or in-hospital death (secondary outcome), we used the multi-state transition model presented in Fig. 1. For this analysis, we reclassified patients who started heparin after PE diagnosis as not treated and, in addition, we performed agin the analysis after having excluded these patients (sensitive analysis). Patients who used intermediate or therapeutic heparin doses were analyzed as a unique group. In the multi-state transition model, we applied the proportional hazards Cox analysis to an extended semi-Markov model with a “clock-forward” time approach, according to Putter et al. [20]. In each state transition, we included variables that predicted PE diagnosis or in-hospital death as assessed previously in the classical Cox multivariate survival model after correction for multicollinearity. Results of Cox analysis were expressed as hazards ratio (HR) with the 95% confidence interval (CI). Sample size was calculated for the primary outcome considering a Cox proportional hazards model. By estimating 20% the prevalence of PE events in COVID-19 patients [2, 4, 21], 0.05 the threshold of type I error, and 2 the hazards ratio for in-hospital death of patients PE exposed with respect to those not exposed [22], we estimated 102 the minimum number of events needed to have at least 80% study’s power. A probability (*p*) lower than 0.050 was significant to exclude the null hypothesis. Statistical analysis was performed with the free software R (version 4.0.1) [23].

## Results

In this study, we included data from 401 consecutive patients of whom general characteristics are reported in Table 1. Patients admitted to the ICU were 19%, the remaining were admitted to sub-intensive or ordinary pulmonary, infectious diseases or internal medicine wards. The median follow-up time of the study was 8 days (range from 1 to 40 days). The most prevalent comorbidities were in order of frequency: hypertension, chronic kidney disease, diabetes, and neoplasia (including solid and hematologic). Patients at admission were equally distributed in the WHO severity categories. The cumulative incidence of PE diagnosis was 27%, and 39% percent of patients with PE received the diagnosis within 24 h from admission. Prevalence of PE diagnosis at admission was 11%. The median time to PE diagnosis was 2 days (IQR 0.5–7). Cumulative mortality was 20% and the median time to death was 12 days (IQR 6–19). At admission, 15.5% patients did not receive heparin treatment, 39.7% received prophylactic dose, and 32.9% received an intermediate or therapeutic dose. All patients after a PE diagnosis started heparin at a therapeutic dose, in particular, 19% of those patients that did not receive heparin at admission and 13% of those that at admission received heparin at a prophylactic dose. Patients that did not receive heparin treatment at all during the study were 9.5%. First prescription of heparin treatment comprised 96% low molecular weight heparin (LMWH), 3% unfractionated heparin, and 1% fondaparinux. Median enoxaparin-equivalent IUs were 4000 (IQR 4000–4000), 6.000 (IQR 6000–8000), and 14,000 (IQR 12,000–16,000) for prophylactic, intermediate, and therapeutic doses, respectively.

**Table 1:** Summary of clinical characteristics and laboratory variables

Clinical characteristic	All patients
Patient (n)	401
Female sex (n (%))	134 (33)
Age (years)	68 ± 13
Body mass index (Kg/m^2^)	28.4 (6.3)
Smoking history (n (%))	48 (12)
COVID-19 severity (WHO category) (n (%))	
• Mild/moderate	150 (37.4)
• Severe	142 (35.4)
• Critical	109 (27.2)
Comorbidities (n (%)):	
• Hypertension	221 (55)
• Chronic kidney disease	110 (27)
• Diabetes	81 (20)
• Active neoplasia	59 (15)
• COPD	25 (6.2)
• Autoimmune disease	23 (5.7)
• Cirrhosis	20 (5.0)
Pulmonary embolism (n (%))	109 (27)
Intensive care unit admission (n (%))	74 (19)
Orotracheal intubation (n (%))	64 (16)
In-hospital death (n (%))	79 (20)
Median follow-up time (days)	8 [5–17]
**Vital sign**	
Body temperature (°C)	36.8 ± 0.7
Respiratory rate (breaths per minute)	21 ± 5
Arterial oxygen saturation (%)	95 [92–97]
Systolic blood pressure (mm Hg)	135 ± 21
Heart rate (bpm)	83 (15)
**Biochemical variable or biomarker**	
Hemoglobin (g/dl)	13.0 ± 1.9
WBC (cells × 10^3^/mm^3^)	7.20 [5.14–10.53]
Lymphocytes (cells × 10^3^/mm^3^)	0.80 [0.50–1.08]
Platelets (cells × 10^3^/mm^3^)	233 [183–321]
CRP (mg/l)	62 [28–111]
D-dimer (ng/dl FEU)	1000 [560–3120]
eGFR (ml/min/1.73m^2^)	78 ± 31
**Drug**	
ACEi/ARB (n (%))	139 (35)
Statin (n (%))	88 (22)
Antiplatelet (n (%))	85 (21)
Any heparin use (n (%))	339 (85)

*PE* Pulmonary embolism, *HR* Hazards ratio, *WHO* World Health Organization, *COPD* Chronic obstructive pulmonary disease, *WBC* White blood cells, *CRP* C-reactive protein, *FEU* Fibrinogen equivalent unit, eGFR Estimated glomerular filtration rate, *ACEi*, Angiotensin converting enzyme inhibitor, *ARB* Angiotensin II receptor blocker

**Fig. 2:**
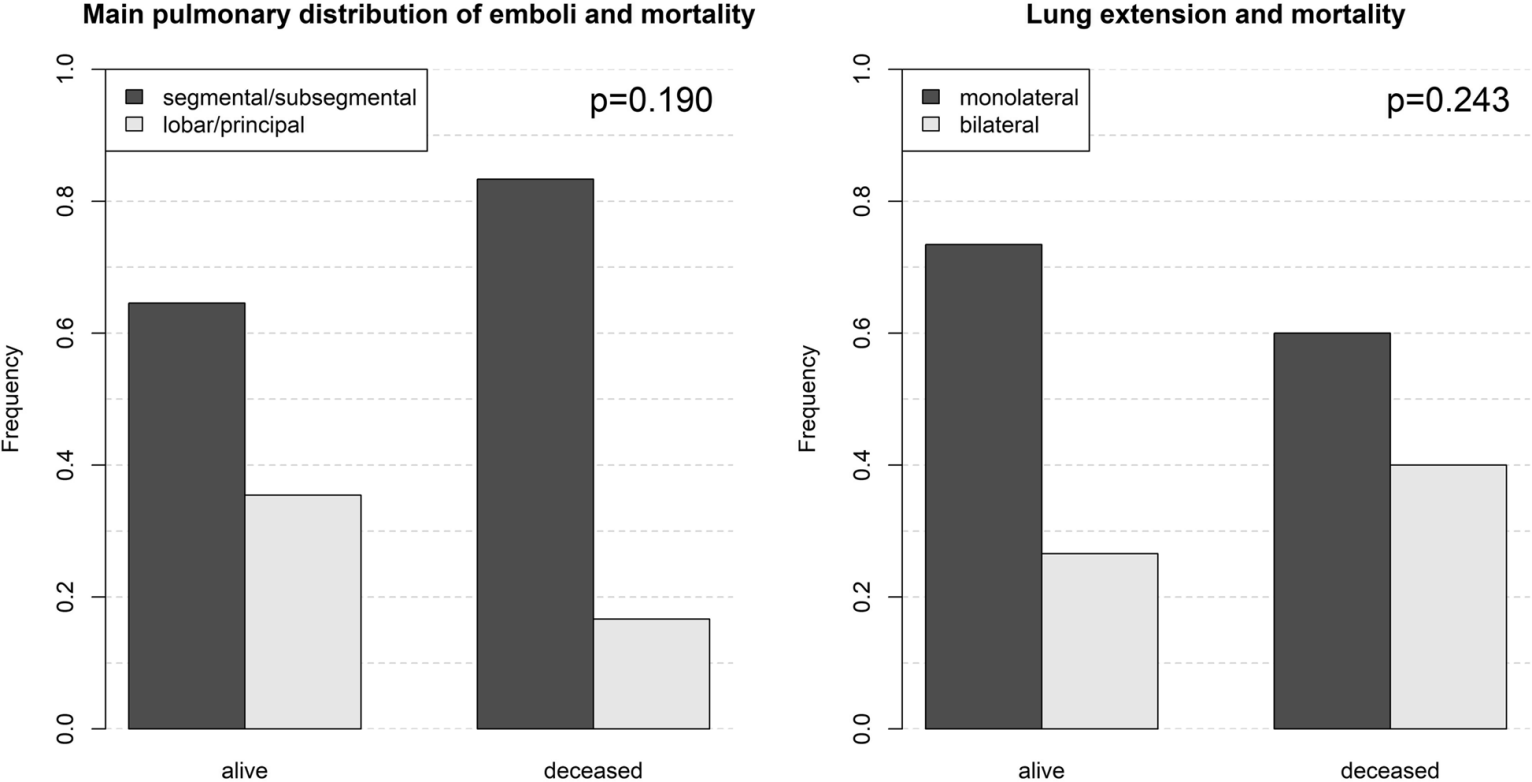
Lung distribution (on the left) and lung extension (on the right) of pulmonary embolism (PE) in radiological imaging in alive and deceased patients at the end of the study

Regarding patients without PE diagnosis, those with PE diagnosis had more severe COVID-19, were admitted more often to ICU, received orotracheal intubation, and had higher mortality. In addition, patients with PE diagnosis had lower body temperature, lower arterial oxygen saturation, greater WBC and platelet count, and greater D-dimer levels (Table 2). Compared to patients who survived, deceased patients were older and more often had critical COVID-19, history of hypertension or active neoplasia, PE diagnosis, and need for orotracheal intubation. In addition, deceased patients had lower BMI, body temperature, arterial oxygen saturation and lymphocytes levels, whereas greater heart rate, WBC, CRP and D-dimer levels (Table 2). Cumulative incidence of PE diagnosis increased across COVID-19 severity WHO class, 10.0%, 36.8%, 38.0%, in mild/moderate, severe, and critical disease class, respectively (*p* < 0.001). The most prevalent anatomical distribution of PE was segmental/sub-segmental, and lung extension was more often monolateral than bilateral. There was no difference in anatomical distribution or lung extension of emboli between alive and deceased patients (Fig. 2).

**Table 2:** Variable description according to PE diagnosis and in-hospital death occurrence

Variable	No PE diagnosis	PE diagnosis	Alive	Deceased
Patient (n)	292	109	322	79
Female sex (n (%))	98 (34)	36 (33)	104 (32)	30 (38)
Age (years)	68 ± 14	70 ± 11	67 ± 13	74 ± 10^c^
Body mass index (Kg/m^2^)	28.4 ± 6.4	28.5 ± 6.1	28.7 ± 6.0	27.2 ± 7.2^a^
Smoking history (n (%))	38 (13)	10 (9.2)	44 (14)	4 (5.1)
COVID-19 (WHO category) (n (%))				
• Mild/moderate	134 (46)	16 (15)^c^	144 (45)	6 (7)^c^
• Severe	91 (31)	51 (47)^b^	125 (39)	17 (22)^b^
• Critical	67 (23)	42 (39)^b^	53 (17)	56 (71)^c^
Comorbidities (n (%)):				
• Hypertension	164 (56)	57 (52)	168 (52)	53 (67)^a^
• Chronic kidney disease	76 (26)	34 (31)	91 (28)	19 (24)
• Diabetes	60 (21)	21 (19)	62 (19)	19 (24)
• Neoplasia	43 (15)	16 (15)	36 (11)	23 (29)^c^
• COPD	20 (6.8)	5 (4.6)	16 (5.0)	9 (11)
• Autoimmune disease	16 (5.5)	7 (6.4)	16 (5.0)	7 (8.9)
• Cirrhosis	16 (5.5)	4 (3.7)	13 (4.0)	7 (8.9)
Pulmonary embolism (n (%))	-	-	79 (25)	30 (38)^a^
Intensive care unit admission (n (%))	33 (11)	41 (38)^c^	40 (12)	34 (43)
Orotracheal intubation (n (%))	29 (10)	35 (32)^c^	31 (10)	33 (42)^c^
In-hospital death (n (%))	49 (17)	30 (32)^a^	-	-
Body temperature (°C)	36.8 ± 0.8	36.6 ± 0.7^a^	36.8 ± 0.8	36.6 ± 0.7^a^
Respiratory rate (breaths per minute)	21 ± 5	21 ± 5	21 ± 5	23 ± 5c
Arterial oxygen saturation (%)	96 [93–98]	94 [91–96]^c^	95 [93–97]	94 [91–96]^b^
Systolic blood pressure (mm Hg)	135 ± 21	137 ± 22	135 ± 21	137 ± 20
Heart rate (bpm)	83 ± 15	84 ± 14	82 ± 14	86 ± 18^a^
Hemoglobin (g/dl)	13.0 ± 1.9	13.0 ± 1.8	13.0 ± 1.8	12.8 ± 2.2
WBC (cells × 10^3^/mm^3^)	6.74 [4.86–10.04]	8.78 [6.17–11.55]^c^	6.84 [5.07–10.00]	9.90 [6.47–12.48]^c^
Lymphocytes (cells × 10^3^/mm^3^)	0.82 [0.58–1.10]	0.64 [0.43–0.93]	0.82 [0.54–1.10]	0.62 [0.38–0.92]^c^
Platelets (cells × 10^3^/mm^3^)	220 [168–299]	265 [209–345]^b^	240 [182–325]	220 [165–295]
CRP (mg/l)	62 [28–111]	66 [32–111]	58 [26–108]	81 [52–121]^c^
D-dimer (ng/ml FEU)	809 [480–2014]	1895 [788–5944]^c^	899 [512–2504]	1555 [673–12300]^c^
eGFR (ml/min/1.73m^2^)	79 ± 32	78 ± 31	78 ± 32	81 ± 31
ACEi/ARB (n (%))	104 (36)	35 (32)	110 (34)	29 (37)
Statin (n (%))	70 (24)	18 (17)	66 (21)	22 (28)
Antiplatelet (n (%))	65 (22)	20 (18)	62 (19)	23 (29)
Any heparin use (n (%))	242 (83)	97 (89)	275 (85)	64 (81)

^a^*p* < 0.050; ^b^*p* < 0.010; ^c^*p* < 0.001

*PE* Pulmonary embolism, *WHO* World Health Organization, *COPD* Chronic obstructive pulmonary disease, *WBC* White blood cells, *CRP* C-reactive protein, *FEU* Fibrinogen equivalent unit, *eGFR* Estimated glomerular filtration rate by MDRD study equation, *ACEi* Angiotensin converting enzyme inhibitor, *ARB* Angiotensin II receptor blocker

**Fig. 3:**
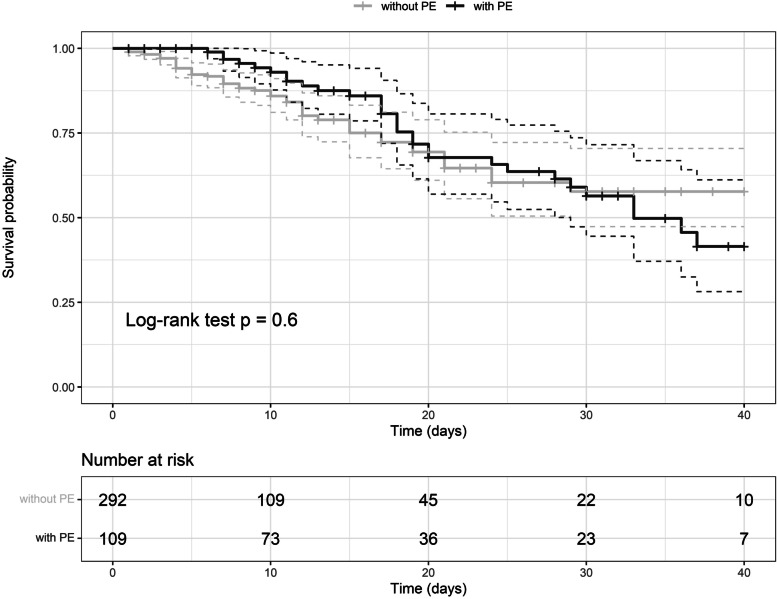
Kaplan–Meier curves representing the survival probability of COVID-19 patients according to having (black line) or not (light gray line) pulmonary embolism (PE) diagnosis. Dashed lines represent the 95% confidence interval range correspondent to the respective color. Below curves, there is the frequency table of patients at risk during the follow-up time

Univariate predictors of PE diagnosis were COVID-19 severity, ICU admission, need for oral intubation, reduced body temperature, reduced arterial oxygen saturation, increased platelet count, and increased D-dimer levels (Table 3). Independent predictors of PE diagnosis in multivariate analysis were COVID-19 severity (severe versus mild/moderate, HR 2.96, 95% CI 1.65–5.29, *p* < 0.001, critical versus mild/moderate HR 2.22, 95% CI 1.21–4.09, *p* = 0.010), decreased body temperature (HR 0.69, 95% CI 0.53–0.90, *p* = 0.007), and increased log D-dimer levels (HR 1.31, 95% CI 1.14–1.51, *p* < 0.001). The variables ICU admission and orotracheal intubation were excluded because they correlated highly with each other and with COVID-19 severity (significant multicollinearity). Univariate predictors of in-hospital death were increased age, lower BMI, critical COVID-19 severity, hypertension, active neoplasia, COPD, increased respiratory and heart rates, reduced platelet count, and increased CRP levels. Independent predictors of in-hospital death (including log D-dimer, PE diagnosis and any heparin use in the multivariate model), were age (HR 1.05, 95% CI 1.03–1.08, *p* < 0.001), body mass index (HR 0.93, 95% CI 0.89–0.98, *p* = 0.004), COVID-19 severity (severe versus mild/moderate HR 3.67, 95% CI 1.30–10.4, *p* = 0.014, critical versus mild/moderate HR 12.1, 95% CI 4.57–32.2, *p* < 0.001), active neoplasia (HR 2.58, 95% CI 1.48–4.50, *p* < 0.001), COPD (HR 2.47; 95% CI 1.15–5.27, *p* = 0.020), respiratory rate (HR 1.06, 95% CI 1.02–1.11, *p* = 0.008), heart rate (HR 1.03, 95% CI 1.01–1.04, *p* < 0.001), and any heparin treatment regarding no treatment (HR 0.35, 95% CI 0.18–0.67, *p* = 0.001). A PE diagnosis did not influence survival probability (Fig. 3).

**Table 3:** Predictors of PE diagnosis and in-hospital death by univariate Cox proportional hazards analysis

Variable	PE HR (95% CI)	*p*	In-hospital death HR (95% CI)	*p*
Patients (n)	109	*-*	79	-
**General clinical characteristics**				
Female sex (no/yes)	0.96 (0.64–1.42)	0.821	1.28 (0.81–2.01)	0.294
Age (each 1 year)	1.01 (0.99–1.02)	0.440	1.04 (1.02–1.07)	< 0.001
Body mass index (each 1 kg/m^2^)	1.01 (0.98–1.04)	0.980	0.95 (0.91–0.99)	0.026
Smoking history (no/yes)	0.72 (0.38–1.39)	0.332	0.37 (0.14–1.02)	0.055
COVID-19 severity:				
• Mild/moderate (Ref.)	1	-	1	-
• Severe	3.63 (2.05–6.45)	< 0.001	2.01 (0.80–5.08)	0.138
• Critical	3.11 (1.72–5.63)	< 0.001	6.33 (2.72–14.76)	< 0.001
Comorbidities (no/yes):				
• Hypertension	0.87 (0.60–1.27)	0.466	1.63 (1.02–2.61)	0.041
• Chronic kidney disease	1.22 (0.81–1.83)	0.248	0.72 (0.42–1.21)	0.213
• Diabetes	0.85 (0.53–1.36)	0.596	1.02 (0.61–1.71)	0.938
• Active neoplasia	0.88 (0.51–1.49)	0.626	1.94 (1.19–3.16)	0.008
• COPD	0.69 (0.28–1.70)	0.421	2.35 (1.17–4.72)	0.016
• Autoimmune disease	1.30 (0.60–2.81)	0.499	2.06 (0.94–4.50)	0.069
• Cirrhosis	0.63 (0.23–1.70)	0.358	1.43 (0.66–3.11)	0.368
Pulmonary embolism (no/yes)	-		0.88 (0.55–1.39)	0.581
ICU admission (no/yes)	2.15 (1.45–3.18)	< 0.001	1.33 (0.84–2.10)	0.231
Orotracheal intubation (no/yes)	1.96 (1.30–2.95)	0.001	1.52 (0.96–2.41)	0.075
**Vital signs**				
Body temperature (each 1 °C)	0.71 (0.53–0.96)	0.024	0.94 (0.70–1.25)	0.667
Respiratory rate (each 1 breath/min)	1.01 (0.97–1.05)	0.601	1.09 (1.04–1.14)	< 0.001
Arterial O_2_ saturation (each 1%)	0.95 (0.91–0.98)	0.001	0.98 (0.93–1.03)	0.340
SBP (each 1 mm Hg)	1.004 (0.995–1.013)	0.160	0.998 (0.988–1.008)	0.671
Heart rate (each 1 bpm)	1.002 (0.990–1.016)	0.369	1.02 (1.00–1.03)	0.012
**Biochemical variable and biomarkers**				
Hemoglobin (each 1 g/dl)	1.04 (0.94–1.14)	0.183	0.95 (0.85–1.07)	0.411
WBC (each 10^3^ cells/mm^3^)	1.02 (0.99–1.05)	0.278	1.02 (0.99–1.06)	0.185
Lymphocytes (each 10^3^ cells/mm^3^)	0.99 (0.93–1.06)	0.897	0.63 (0.34–1.17)	0.148
Platelets (each 10^4^ cells/mm^3^)	1.02 (1.01–1.04)	0.006	0.97 (0.95–0.99)	0.010
CRP (each 1 log mg/l)	1.08 (0.90–1.29)	0.193	1.41 (1.09–1.83)	0.009
D-dimer (each 1 log ng/dl FEU)	1.32 (1.17–1.50)	< 0.001	1.10 (0.93–1.29)	0.258
eGFR (each 1 ml/min/1.73m^2^)	0.99 (0.99–1.01)	0.672	1.002 (0.996–1.009)	0.500
**Drugs**				
ACEi/ARB (no/yes)	0.81 (0.54–1.22)	0.315	1.07 (0.68–1.69)	0.769
Statin (no/yes)	0.64 (0.38–1.06)	0.080	1.63 (0.99–2.67)	0.053
Antiplatelet (no/yes)	0.75 (0.46–1.21)	0.235	1.48 (0.91–2.40)	0.116
Any heparin use (no/yes)	1.23 (0.68–2.25)	0.496	0.59 (0.34–1.04)	0.068

*PE* Pulmonary embolism, *HR* Hazards ratio, *WHO* World Health Organization, *COPD* Chronic obstructive pulmonary disease, *WBC* White blood cells, *CRP* C-reactive protein, *FEU* Fibrinogen equivalent unit, *eGFR* Estimated glomerular filtration rate, *ACEi* Angiotensin converting enzyme inhibitor, *ARB* Angiotensin II receptor blocker

To account for the time-dependent effect of preventive heparin treatment on PE diagnosis and in-hospital death, we considered the multi-state transition model reported in Fig. 1 after having reclassified patients who started heparin after PE diagnosis as not treated. With this reclassification, PE was diagnosed in 50%, 13%, and 23%, of patients with none, prophylactic, and intermediate/therapeutic preventive heparin treatment, respectively (*p* < 0.001). Positive and negative independent predictors of PE diagnosis and in-hospital death were reported for each state transition in Table 4. Positive predictors of transition 1 (PE diagnosis), were COVID-19 severity and log D-dimer levels, whereas the only negative predictor was preventive heparin treatment at any dose regarding no treatment. Positive predictors of transition 2 (death without a PE diagnosis) were age, COVID-19 severity, and active neoplasia, whereas the only negative predictor was preventive heparin treatment at any dose regarding no treatment. Positive predictors of transition 3 (death after a PE diagnosis) were active neoplasia and COVID-19 severity. Preventive heparin did not show any effect on this last state transition. We neither observed an effect of PE diagnosis nor of the time to PE diagnosis on in-hospital death (Table 4). The sensitive analysis was conducted in 342 patients after having excluded 59 patients who had started heparin after PE diagnosis. In this subgroup, PE was diagnosed in 61 patients (state transition 1) and death occurred in 49 patients without PE diagnosis (state transition 2) and in 23 patients after PE diagnosis (state transition 3). The result of the sensitive analysis confirmed that of the whole sample with patients’ reclassification (Table 4).

**Table 4:** Effect of preventive heparin on PE diagnosis and in-hospital death by multi-state transition model

Variable	Patients who started heparin after PE diagnosis reclassified as not treated (*n*=401)		Patients who started heparin after PE diagnosis are excluded from the analysis (*n*=342)	
	**HR (95% CI)**	***p***	**HR (95% CI)**	***p***
**Transition 1 (COVID-19 Dx → PE)**				
Age (each year)	1.00 (0.98–1.01)	0.600	0.99 (0.97–1.02)	0.659
BMI (each 1 kg/m^2^)	1.00 (0.97–1.03)	0.992	1.00 (0.96–1.04)	0.928
COVID-19 severity (each)	1.56 (1.21–2.01)	< 0.001	1.43 (1.00–2.04)	0.050
Active neoplasia (no/yes)	0.83 (0.48–1.45)	0.513	0.77 (0.35–1.71)	0.523
COPD (no/yes)	0.50 (0.20–1.25)	0.140	0.39 (0.09–1.63)	0.195
D-dimer (log ng/dl FEU)	1.34 (1.17–1.54)	< 0.001	1.40 (1.17–1.68)	< 0.001
Preventive heparin use:				
• No (Ref.)	1	-	1	-
• Prophylactic dose	0.13 (0.07–0.22)	< 0.001	0.29 (0.14–0.63)	0.002
• Intermediate/therapeutic dose	0.18 (0.11–0.28)	< 0.001	0.41 (0.19–0.86)	0.018
**Transition 2 (COVID-19 Dx → DEATH)**				
Age (each year)	1.08 (1.05–1.12)	< 0.001	1.07 (1.04–1.11)	< 0.001
BMI (each 1 kg/m^2^)	0.94 (0.89–1.00)	0.062	0.94 (0.89–1.00)	0.068
COVID-19 severity (each 1 class)	3.20 (2.08–4.92)	< 0.001	3.31 (2.16–5.06)	< 0.001
Active neoplasia (no/yes)	2.66 (1.35–5.24)	0.005	2.55 (1.30–5.02)	0.007
COPD (no/yes)	1.98 (0.87–4.50)	0.101	1.73 (0.76–3.95)	0.193
D-dimer (log ng/dl FEU)	0.86 (0.69–1.08)	0.196	0.88 (0.71–1.11)	0.284
Preventive heparin use:				
• No (Ref.)	1	-	1	-
• Prophylactic dose	0.43 (0.20–0.92)	0.029	0.31 (0.14–0.68)	0.003
• Intermediate/therapeutic dose	0.44 (0.19–0.97)	0.042	0.30 (0.13–0.71)	0.006
**Transition 3 (PE → DEATH)**				
Age (each year)	1.01 (0.97–1.05)	0.644	1.01 (0.96–1.05)	0.721
BMI (each 1 kg/m^2^)	0.99 (0.94–1.04)	0.649	0.99 (0.94–1.05)	0.822
COVID-19 severity (each 1 class)	3.74 (1.59–8.80)	0.002	3.28 (1.21–8.91)	0.019
Active neoplasia (no/yes)	3.92 (1.45–10.5)	0.007	3.80 (0.89–16.3)	0.072
COPD (no/yes)	0.73 (0.14–3.79)	0.712	0.85 (0.12–6.05)	0.870
D-dimer (log ng/dl FEU)	1.03 (0.76–1.39)	0.856	0.98 (0.67–1.44)	0.932
Preventive heparin use:				
• No (Ref.)	1	-	1	-
• Prophylactic dose	1.37 (0.49–3.83)	0.549	1.16 (0.23–7.89)	0.857
• Intermediate/therapeutic dose	1.43 (0.53–3.85)	0.474	1.12 (0.22–5.72)	0.893
**Effect of PE diagnosis and time to PE diagnosis on in-hospital death**				
PE diagnosis (no/yes)	2.3 (0.00–1003)	0.792	2.2 (0.00–2006)	0.822
Time to PE diagnosis (each day)	1.06 (0.99–1.13)	0.086	1.07 (0.98–1.17)	0.113

*HR* Hazards ratio, *PE* Pulmonary embolism *WHO* World Health Organization, *BMI* Body mass index, *COPD* Chronic obstructive pulmonary disease, *Ref.* Reference, *FEU* Fibrinogen equivalent units

## Discussion

In this study, we confirmed the high incidence of PE diagnosis in hospitalized COVID-19 patients [4, 21]. Suh et al. in a meta-analysis of 27 observational studies, showed a pooled incidence of PE in COVID-19 patients admitted to medical wards and ICU of 17% (95% CI 12–23), with the highest value in ICU admitted patients (25%, 95% CI 19–32) [4]. In our study, cumulative incidence of PE was consistent with that observed by Suh et al. in critical ICU patients, despite only one-fifth of our patients were ICU admitted. This can be reasonable because about two-thirds of our patients showed at admission a severe/critical disease but, since ICU overcrowding, many of these patients were managed initially in a sub-intensive medical ward. Therefore, the cumulative incidence of PE that we observed reflects the high prevalence of severe/critical patients rather than the type of ward of first admission, and comparing our results with that of other studies based on the type of admission could be misleading. In addition, we observed a prevalence of PE diagnosis at admission of 11%, a proportion very similar to that observed by Jevnikar et al. [24]. Mostly of these diagnoses occurred incidentally because of a CT scan performed for COVID-19 diagnostic work-up. These findings suggest that PE is common in not anticoagulated COVID-19 patients and that COVID-19 is a prothrombotic condition.

The activation of the hemostatic system characterizes the prothrombotic state of COVID-19, of which plasma D-dimer concentration is a sensitive marker [25, 26]. Retrospective and prospective studies have shown a strong positive correlation between D-dimer levels and PE occurrence [27, 28]. In our study, increased D-dimer levels predicted PE diagnosis in the multivariate model, thus confirming its importance as an independent risk factor for PE. A D-dimer level higher than 500 ng/dl have shown an elevated sensitivity (96%, 95% CI 93–97) and negative predictive value (88%, 95% CI 78–97), but a low specificity (10%, 95% CI 7–14) and positive predictive value (26%, 95% CI 25–27) in predicting PE diagnosis [4]. Elevated D-dimer has been associated also with COVID-19 severity and mortality independently of PE occurrence [14, 29]. These findings suggest that although PE can be detected in a higher than expected proportion of COVID-19 patients with elevated D-dimer, D-dimer alone is more useful for excluding rather than suggesting a PE diagnosis. In our study, heparin was effective to reduce mortality independently of both PE diagnosis and D-dimer levels. Therefore, getting preliminary D-dimer levels or looking for a PE diagnosis by lung imaging appeared not essential to start anticoagulation in hospitalized COVID-19 patients.

In our cohort, PE was more frequent in severe/critical and deceased patients, but PE diagnosis did not predict mortality in both Cox classical survival and multi-state models. Other observational studies in severe and critical patients reported similar results [13, 30]. This can be reasonable if we consider most of PE in COVID-19 patients without overt DVT as a marker of disease severity rather than a severe VTE complication [31]. Lung imaging of COVID-19 patients commonly showed the involvement of segmental and sub-segmental vessels that, in pathological studies, have been associated most times with localized pulmonary thrombosis [28, 32]. In addition, autopsies of COVID-19 patients showed pulmonary thrombosis that was asymptomatic and not diagnosed in-vivo [2]. Pulmonary thrombosis is probably the consequence of inflammation, hypoxia, and endothelial damage because of SARS-CoV-2 lung infection [33, 34]. Pulmonary thrombosis in COVID-19 patients has been associated with a more severe disease and respiratory complications [35]. Therefore, the meaning of PE detected by lung imaging in COVID-19 patients without overt DVT might be a sign of lung involvement [36] and should be considered more properly as a marker of disease severity [37]. The role of anticoagulation or thrombolysis on pulmonary thrombosis in COVID-19 patients remains uncertain [38].

Observational studies showed that anticoagulation therapy improved survival of COVID-19 patients [21, 39] and this is in line with our findings. However, we observed also that although heparin treatment prevented PE diagnosis, PE occurred despite thromboprophylaxis in some patients and, in these patients, preventive heparin did not change mortality risk. This last finding could be justified by the low number of death in this group of patients. However, more considerations on this point can be done. The lack of a benefit of preventive heparin on mortality in patients with a PE diagnosis might be related to the fact that all these patients started heparin at a therapeutic dose after PE diagnosis. In addition, the occurrence of PE despite preventive heparin suggests that these patients might present a resistance to the heparin effect [40, 41] or that the potential bleeding risk associated with higher than prophylactic doses of heparin might have overwhelmed the protective effect of anticoagulation on mortality [10, 42]. On this last point, an important limit of this study was not having included information about arterial thrombotic events and major bleedings. Both these events can impact on mortality and are influenced by heparin treatment, so that their occurrence could have affected the mortality risk of our patients. Unfortunately, this point remains unclear and needs more evidence.

Another independent predictor of in-hospital mortality in this study was a low BMI. To our knowledge, this association known as “obesity paradox”, although it has been showed in other serious infection and in critically ill patients, it has not been recognized in COVID-19 patients, in which obesity was consistently associated with increased mortality [43]. Therefore, this observation should be considered cautiously and more studies are needed to clarifying this point.

This study presented some limitations to discuss. First, the number of events needed for the primary outcome was not reached. This occurred probably because of the misestimation of PE prevalence and mortality in COVID-19 patients. The hazards ratio for mortality regarding patients exposed to or not to PE in this study was lower than that expected. This could have been determined by the misinterpretation of PE detected by lung imaging in COVID-19 patients without overt DVT in previous studies. Second, in this study, PE was screened at admission and at the time of symptoms that suggested PE diagnosis. However, because PE can occur also asymptomatic, we could have missed some PE events and underestimated its prognostic role. Third, the use of heparin during the study period was heterogeneous, because of the heterogeneity of specialists who have followed COVID-19 patients and the lack of a common thromboprophylaxis protocol. In addition, some patients changed doses and type of anticoagulants according to changes in their clinical conditions. Therefore, there were many clusters of different heparin-treated patients with an elevated risk of selection bias. To reduce this problem, we performed multivariate analysis, including all measured confounders, and made our results consistent by sensitive analysis. Fourth, this retrospective study involved patients unvaccinated for SARS-CoV-2 during the first waves of infection and before results of clinical trials on thromboprophylaxis were available. Therefore, our findings cannot be extrapolated to the present COVID-19 situation where patients were widely vaccinated and antithrombotic protocol were adopted for hospitalized patients.

## Conclusions

This study shows that PE was not a prognostic factor for mortality in COVID-19 patients without overt DVT. Heparin treatment at any dose compared to no treatment predicted a low mortality rate independent of PE diagnosis, D-dimer levels, and disease severity. Preventive heparin treatment reduced the risk of PE diagnosis, but it did not reduce mortality in patients with a PE diagnosis. Our results confirmed the usefulness of heparin for reducing mortality of unvaccinated COVID-19 patients during the first waves pandemic.

## Data Availability

The datasets analyzed during the current study are available from the corresponding author on reasonable request and Institutional authorization.

## Abbreviations

SARS-CoV-2: Severe acute respiratory syndrome coronavirus 2
COVID-19: Coronavirus 2019 induced disease
PE: Pulmonary embolism
DVT: Deep vein thrombosis
IU: International unit
BMI: Body mass index
HR: Hazards ratio
WHO: World Health Organization
RT-PCR: Real-time reverse transcriptase polymerase chain reaction
ICU: Intensive care unit
COPD: Chronic obstructive pulmonary disease
WBC: White blood cells
CRP: C-reactive protein
FEU: Fibrinogen equivalent unit
MDRD: Modification of Diet in Renal Disease
eGFR: Estimated glomerular filtration rate
ACEi: Angiotensin converting enzyme inhibitor
ARB: Angiotensin II receptor blocker
CI: Confidence interval
